# Well-being variations on students of health sciences related to their learning opportunities, resources, and daily activities in an online and on-crisis context: a survey-based study

**DOI:** 10.1186/s12909-023-04011-y

**Published:** 2023-01-18

**Authors:** Cristhian Pérez-Villalobos, Juan Ventura-Ventura, Camila Spormann-Romeri, Ximena Paredes-Villarroel, Marcos Rojas-Pino, Catherine Jara-Reyes, Mildred Lopez, Isidora Castillo-Rabanal, Mary Jane Schilling-Norman, Marjorie Baquedano-Rodríguez, Paula Parra-Ponce, Josselinne Toirkens-Niklitschek, Juan Carlos Briede-Westermeyer, Débora Alvarado-Figueroa

**Affiliations:** 1 Medical Education Department, School of Medicine, Universidad de Concepción, Concepción, Chile; 2 Medical Technology Department, School of Medicine, Universidad de Tarapacá, Arica, Chile; 3 Coordination of educational management in health (CGES), Department of Health, Universidad de Los Lagos, Osorno, Chile; 4 Department of Health Sciences, Universidad de Aysén, Coyhaique, Chile; 5 Graduate School of Education, Stanford University, Stanford, USA; 6 Biomedical Department, Faculty of Health Sciences, Universidad de Antofagasta, Antofagasta, Chile; 7 Escuela de Medicina y Ciencias de la Salud, Tecnologico de Monterrey, Monterrey, Mexico; 8 University College London, London, UK; 9 Academic and Social-Emotional Accompaniment Directorate, Universidad Catolica de Temuco, Temuco, Chile; 10 Department of Engineering Design, Universidad Técnica Federico Santa María, Valparaíso, Chile

**Keywords:** Online education, Social support, Pandemics, Medical students, Online teaching, E-learning, COVID-19, Structural equation modeling

## Abstract

**Background:**

Universities’ training process intensely relies on face-to-face education. The COVID-19 pandemic interrupted it and forced them to reinvent their process online. But this crisis seems not to be the last we will face, and we take it as a lesson to prepare for future crises. These critical contexts are especially challenging because they imply changing teaching strategies, and students may not have the technology access or the living conditions to connect as they need. They also lived through a pandemic where the virus and the life changes added stress to their learning process and threatened their well-being. So, this study aims to analyze how well-being variations reported by Health sciences students relate to their learning opportunities, access conditions, and daily activities.

**Method:**

We surveyed 910 Health sciences students from six different Chilean universities at the end of the first semester of 2020, the first in pandemic conditions. Respondents answered online questionnaires about 1) Remote teaching activities, 2) Learning resources availability, 3) Daily life activities, and 4) Well-being changes. We performed descriptive analysis and Structural Equation Modelling.

**Results:**

Live videoconference classes were the most frequent teaching activity; only a third of the students had quiet spaces to study online, and most had to housekeep daily. More than two third reported some well-being deterioration. The structural equation model showed a good fit.

**Conclusion:**

Results show an online learning scenario that tries to emulate traditional learning focusing on expositive strategies. Most students reported that their well-being deteriorated during the semester, but tutorials, workplace availability, and social support were protective factors.

**Supplementary Information:**

The online version contains supplementary material available at 10.1186/s12909-023-04011-y.

## Background

The year 2020 swept away the traditional concept of what university is and forced us to reinvent it online. Now, understanding how the COVID-19 crisis affected the educational process is a pivotal opportunity to prepare for future uncertain crisis environments. As a world crisis, the COVID-19 pandemic forced mobility restrictions to diminish the spread of the virus [1]. Since World War II, no other disruption, but this pandemic has impacted face-to-face education to the point of closing the campus doors and to transit to remote emergency teaching [2, 3]. Developed countries led this transition [4].

It is not that educational institutions in underdeveloped countries did not want to offer quality education for their learners, but this crisis shocked them. The problem with this rapid transition is that universities are complex organizations that have developed mechanisms, systems, infrastructure, and teams dedicated to supporting them for centuries. Universities sought to strengthen their educational function through the continuous evolution of study rooms, academic records, food services, support units for students and faculties, and even computer resources. Albeit this evolution could seem slow for centers that must be knowledge managers. However, these means relied heavily on face-to-face delivery, and this could no longer be. To survive, universities needed to adapt and rethink their mechanisms to thrive in an online setting. Hodges refers to this process as emergency remote teaching, a transition to remote teaching distinguished from online education by its abruptness, lack of planning, and emphasis on maintaining service over quality [2].

Thus, teachers had to devise new ways of delivering learning resources, although often, time constraints and the stress of the pandemic meant replicating traditional strategies with little innovation and creativity [5]. Some educators intensely relied on methods such as videoconferencing [6]. Although students also expected traditional strategies to be maintained [7], adapting to online teaching caused them anxiety [8] and stress [9]. Some students overturn their prejudices about it [10], and several studies show that students have ended up at least moderately satisfied with the process and with the learning achieved [11–15]. Nevertheless, several still prefer face-to-face learning [11, 15].

However, limited access to technologies and continuous Internet access are a constant concern [6, 11–13]. And it mainly affects low-income groups that possess less technology and have more problems learning to use them [16]. In this sense, the crisis did not affect the entire population equally, as people belonging to minority groups in poverty or from other marginalized groups were particularly affected [17]. Learners have not been an exception.

Nevertheless, emergency remote teaching seems to mask a more complex process. Although universities have emphasized academic continuity to continue the training at all costs [3, 18], it was not only teaching that migrated abruptly. So did most institutional mechanisms, such as student support and counseling; we have witnessed the rise of a true Emergency Remote University. Society, even though it has been deeply affected by a pandemic for more than two years, is still present in 2022, generating health, economic and socio-cultural consequences that we must assess in the years to come [3, 14].

The pandemic strongly affected students’ work-life balance [19], and relates to feelings of distress or preoccupation with academic duties, isolation, and even discrimination [10]. Mental health problems increased during the pandemic, especially among individuals between 18 and 24 years old, who were the most affected [20]. Coincidently, this age group is where most university students are. From research, we know that the occurrence of mental health problems among students during the pandemic may be associated with the anxiety of having the virus around them, changes in the family economy, and access to protective measures [21]. However, emergency remote teaching also plays a role, as students less satisfied with it tend to more frequently present stress, anxiety, and depression [22].

The Latin American region received the most brutal hit in the second wave of the virus. In this zone, these contextual factors have been determinants in access to quality education, clean water, and other healthy living lifestyles that are rights taken for granted in developed countries. It is impossible to design effective teaching without understanding the context in which students live and making deliberate efforts to know their conditions [16]. As early as 1987, Shulman indicated that knowledge of the context was one of the three pillars of didactic knowledge of content, together with disciplinary and pedagogical knowledge [23].

For this reason, the present study first describes the conditions of undergraduate Health sciences students in Chile during the first semester of the pandemic. It presents a Structural Equation Model that analyzes how the variations in the students’ well-being were associated with the teaching respondents experienced as learning opportunities, their access to learning resources, and their daily activities.

## Methods

### Ethics statement

This research followed the guidelines and regulations of the Declaration of Helsinki and Chilean law 20,102 about scientific research in human beings.

All study participants were above 18 years old and gave informed consent to participate in this research.

This research obtained ethical approval from the Ethical Science Committee of the Faculty of Medicine of Universidad de Concepción, Chile, the institution responsible for the project (No. CEC 102020). The rest of the participating universities accepted this approval to authorize the study.

### Study design

It is a survey-based descriptive study. We carried out a quantitative research approach using a non-experimental, cross-sectional, and analytic design.

### Setting

**Fig. 1:**
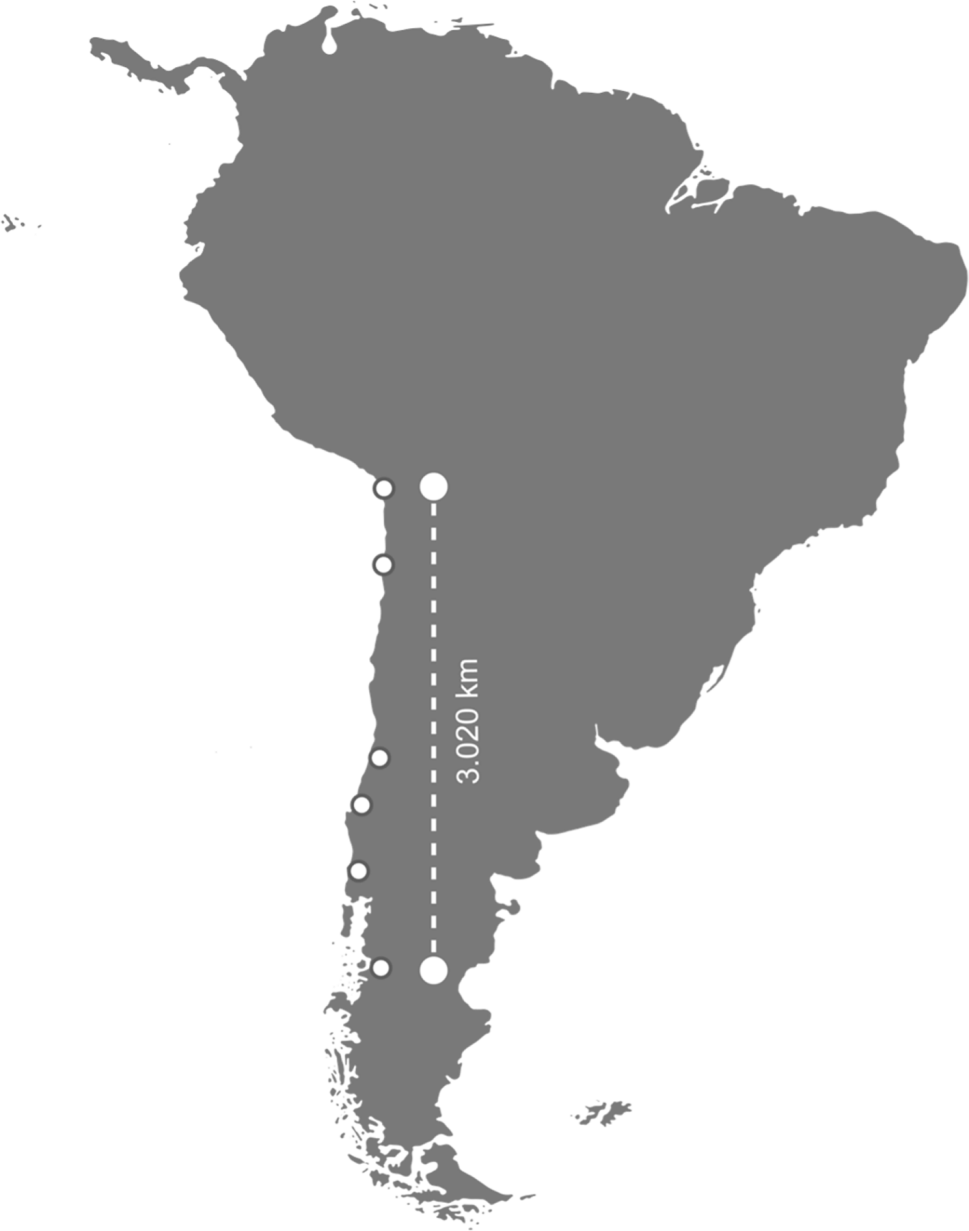
The approximate geographical location of the participating universities in South America

This study included six Chilean universities selected to obtain a representative geographical area of this country. These educational institutions spread over 3020 km from the northernmost to the southernmost one, representing six different cities: Arica, Antofagasta, Santiago, Concepción, Osorno, and Coyhaique, Fig. 1.

All these institutions are financed by the Chilean government.

We performed this research in 2020 at the beginning of the COVID-19 pandemic. As the Chilean academic year lasts from March to December, and the confinement started in this country in March 2020, the 2020 academic year was carried out entirely online.

We collected data during the third and fourth months of the first academic term of 2020.

### Participants

The number of all target students was 9365 students. We calculated a minimum sample size of 190 participants in order to detect a medium effect size (0.3), considering a power of 0.8 and a confidence interval of 95% for a structural equation model of 42 observed variables and ten latent variables. The obtained sample consisted of 910 Health sciences students using non-probabilistic volunteer sampling: All of the 9365 students from the studied schools were invited to participate and chose if they wanted to be involved in the study through a self-selection process. Respondents’ average age was 21.07 years (SD = 3.16), and 78.7% were women, representing seven different undergraduate programs in their six universities, Table 1.

**Table 1:** Description of the surveyed health students

Variable	Values	n; %
Gender	Women	724; 79.6%
	Men	186; 20.4%
University	University 1	355; 39.0%
	University 2	49; 5.4%
	University 3	201; 22.1%
	University 4	66; 7.3%
	University 5	172; 18.9%
	University 6	67; 7.4%
Degree	Medical technology	105; 11.5%
	Medicine	143; 15.7%
	Midwifery	179; 19.7%
	Nursing	227; 24.9%
	Kinesiology	120; 13.2%
	Speech therapy	77; 8.5%
	Nutrition and dietetics	59; 6.5%
Level	First year	279; 30.7%
	Second year	184; 20.2%
	Third year	191; 21.0%
	Fourth year	184; 20.2%
	Fifth year	51; 5.6%
	Sixth year	8; 0.9%
	Seventh year	13; 1.4%

**Table 2:** Remote Teaching Activities during the first academic term of 2020 as reported by students

	Never		Almost never		Sometimes		Almost always		Always	
	n	%	n	%	n	%	n	%	n	%
Videoconference classes	16	1.8	42	4.6	141	15.5	322	35.4	389	42.7
Narrated classes > 30 min	98	10.8	117	12.9	253	27.8	233	25.6	209	23.0
Narrated classes < 30 min	230	25.3	258	28.4	267	29.3	114	12.5	41	4.5
Tutorial videos	337	37.0	239	26.3	247	27.1	70	7.7	17	1.9
Videos about experiences or stories	391	43.0	222	24.4	214	23.5	61	6.7	22	2.4
Infografics	177	19.5	225	24.7	308	33.8	137	15.1	63	6.9
Submission of texts with subsequent discussion	97	10.7	134	14.7	260	28.6	251	27.6	168	18.5
Submission of texts with subsequent activity	124	13.6	189	20.8	278	30.5	214	23.5	105	11.5
Submission of texts without subsequent activity	122	13.4	186	20.4	266	29.2	211	23.2	125	13.7
Forums	101	11.1	138	15.2	274	30.1	193	21.2	204	22.4
Practical exercises	131	14.4	178	19.6	305	33.5	201	22.1	95	10.4
Group work	36	4.0	80	8.8	244	26.8	316	34.7	234	25.7
Tutorials with teacher (< 10 students)	429	47.1	169	18.6	181	19.9	95	10.4	36	4.0
Tutorials with teacher (10 to 20 students)	374	41.1	166	18.2	178	19.6	140	15.4	52	5.7
Individual tutoring meeting	776	85.3	76	8.4	37	4.1	15	1.6	6	0.7

### Variables

This study considered seven variables. Below we describe its operational definitions:

1) Remote Teaching Activities: Assessed by the four scores of the Remote Teaching Activities Questionnaire: Expository activities, Planned Digital Resources, Interactive Activities, and Tutorials.

2) Learning Resources Availability: Assessed by the two Learning Resources Availability Questionnaire scores: Workplace availability and Technology resources.

3) Daily Life Activities: Measured by the two scores of the Daily Life Activities Questionnaire: Housekeeping and Caring for others.

4) Well-being changes: Measured by the two scores of the Well-being Changes Questionnaire: Well-being and Social Support.

5) Gender: Evaluated from the students’ response to the question “Gender” with three options: Male and Female.

6) Age: Measured by the number of years students reported in the “Age” question.

7) Level: Evaluated from the level (from first to seventh) that students gave to the question: “Considering most of the courses you are taking this semester, what level of your degree are you taking?”

### Data collection

The students answered a questionnaire set that included the following:

1) Remote Teaching Activities Questionnaire: A 16-items questionnaire that asked how frequently the students experienced different teaching activities during the first term of 2020. It used a five-point scale that ranged from 0 (never) to 4 (always): 0 = Never, 1 = Almost never, 2 = Sometimes, 3 = Almost always, and 4 = Always). According to McDonald’s Omega, this study showed a reliability of *ω* = 0.63 for Expository activities, *ω* = 0.66 for Planned Digital Resources, *ω* = 0.67 for Interactive Activities, and *ω* = 0.75 for Tutorials.

2) Learning Resources Availability Questionnaire: This questionnaire asked students how often they resorted to learning resources during online courses, which ranged from quiet study places to technology equipment. Students had six choices to respond: 0 = Never, 1 = At least once a month, 2 = At least once a week, 3 = A few days a week, 4 = At least once a day, and 5 = Whenever required. Its reliability was *ω* = 0.93 for Workplace availability and *ω* = 0.87 for Technology resources.

3) Daily Life Activities Questionnaire: To identify how frequently the students had to perform daily life activities regarding work, cooking, housekeeping, or caring for others. Respondents had to mark one of five options: 0 = Never; 1 = Few times a month; 2 = At least once a week, 3 = Several times a week, or 4 = Every day. Its reliability was *ω* = 0.72 for Housekeeping and *ω* = 0.75 for Caring for others.

4) Well-being Changes Questionnaire: Finally, we ask them about the changes in different aspects of personal well-being since the emergency remote teaching term started. It included variations in stress, workload, perceived well-being, emotional stability, sleep quality, eating pattern quality, family support, social support, and studies-life balance. Students evaluated and identified the personal changes in these aspects, choosing among seven alternatives: − 3 = Radically worsened, − 2 = Much worsened, − 1 = Somewhat worsened, 0 = Maintained equal, 1 = Somewhat improved, 2 = Much improved, 3 = Radically improved. Its reliability was *ω* = 0.75 for Well-being and *ω* = 0.93 for Social Support.

The research team created all the questionnaires for this study, and then we sent them to a purposive sample of 12 psychology, education, and medical education experts to assess the measures’ content validity. In order to do this, experts received a form with each variable’s conceptual definition and a list of proposed items and response choices. Then, experts rated them according to the contribution of each item to measure the corresponding variable. We calculated each item’s content validity ratio (CVR) and retained those with scores above CVR = 0.62. Before applying to the final sample, we performed a pilot application to 15 students and a cognitive interview to identify aspects to improve.

### Procedure

The participant universities gave their institutional approval before the data collection started. Then we requested the authorized head of each Health sciences school to distribute an e-mail to the institutional e-mail students’ addresses inviting them to complete the questionnaire set. After 15 days, we re-send the invitation to those who did not respond to the survey the first time. This e-mail linked them to an online survey on the SurveyMonkey© platform, which commenced with an informed consent form. If participants gave their consent, the survey drove them to answer the questionnaires, but the survey immediately closed for them if they refused.

### Data analysis

First, we performed a descriptive data analysis of every item in each of the four questionnaires showing a detailed approach to students’ experiences in the evaluated variables.

Finally, for testing our conceptual model, we carried out a structural equation modeling method using the Weighted Least Square Mean and Variance adjusted (WLSMV) estimator because the included observed variables were categorical. We used as model fit indicators the following: χ^2^-test, which is widely used but is known for its tendency to reject lightly miss-specified models [24, 25]; the χ2/df ratio, the Comparative Fit Index (CFI), the Tucker-Lewis Index (TLI), the Root Mean Square Error of Approximation (RMSEA) and its 90% confidence interval (CI), and Standardized Root Mean-Square Residual (SRMR). We considered χ2/df < 5 for an acceptable fit, χ2/df < 3 for a good fit, and χ2/df < 2 when it was excellent. CFI ≥ 0.95, TLI ≥ 0.95, RMSEA< 0.06, and SRMR< 0.08 were considered cut-off values of a good fit, and CFI ≥ 0.90, TLI ≥ 0.90, RMSEA< 0.10, and SRMR< 0.12 when the fit was acceptable [26–29]. Mplus 8.6 was the software used for data analysis.

## Results

### Students’ experiences during their online learning

First, we analyzed the frequency with which students reported having experienced different remote teaching activities as learning opportunities. As a result, the most frequent teaching activities were live videoconference classes (42.7% of the students said these classes were “always” used, and another 35.4% reported that they were used “almost always.” The following more frequent activities were group assignments (25.7% reported as “always”), forums (22.4% reported as “always”), and narrated lecture videos of more than 30 minutes (23.0% reported as “always”). The most atypical activity was the individual tutoring meeting which never occurred, according to 85.3% of the students, Table 2.

In terms of learning resources used, the most widely available were the smartphone (94.4%), the Internet for social media (83.2%), and computer (80.8%), which were accessible every time students needed them. These resources were followed by access to cameras for videoconferencing (74.0%), microphones for videoconferencing (73.1%), and broadband Internet (73.1%). It is worth noting that a resource of tremendous importance, such as a quiet space to study, was only available when required for 29.0% of the students, and only 34.0% had that access to places for participating in videoconferences, Table 3.

**Table 3:** Availability of learning resources during the first academic term of 2020 as reported by students

	Never		At least once a month		At least once a week		A few days each week		At least once a day		Whenever required	
	n	%	n	%	n	%	n	%	n	%	n	%
Quiet spaces to study	76	8.4	28	3.1	92	10.1	281	30.9	169	18.6	264	29.0
Quiet places for videoconferencing	79	8.7	36	4.0	77	8.5	225	24.7	184	20.2	309	34.0
Broadband Internet access	36	4.0	6	0.7	9	1.0	63	6.9	131	14.4	665	73.1
Internet access for social media	4	0.4	2	0.2	7	0.8	34	3.7	106	11.6	757	83.2
Computer	24	2.6	5	0.5	11	1.2	57	6.3	78	8.6	735	80.8
Tablet	640	70.3	4	0.4	8	0.9	12	1.3	16	1.8	230	25.3
Smartphone	10	1.1	3	0.3	3	0.3	10	1.1	25	2.7	859	94.4
Microphone for videoconferencing	98	10.8	9	1.0	17	1.9	57	6.3	64	7.0	665	73.1
Camara for videoconferencing	94	10.3	9	1.0	23	2.5	53	5.8	68	6.4	673	74.0
Camara for videorecording	229	25.2	13	1.4	21	2.3	45	4.9	41	4.5	561	61.6

During the pandemic, studying from home was not the only occupation for students. Participants also need to perform other daily tasks; for example, students reported they had to clean the house (33.4%) or cook (21.1%) daily. In addition to caring for other people: 18.1% of the students cared for minors daily, 6.7% cared for the elderly, and 4.6% cared for sick people, Table 4.

**Table 4:** Daily Life Activities during the first academic term of 2020 as reported by students

	Never		Few times by month		At least once a week		Some days each week		Every day	
	n	%	n	%	n	%	n	%	n	%
Cooking	82	9.0	166	18.2	183	20.1	287	31.5	192	21.1
House cleaning	32	3.5	82	9.0	192	21.1	300	33.0	304	33.4
Caring for minors	456	50.1	105	11.5	77	8.5	107	11.8	165	18.1
Caring for the older adults	694	76.3	72	7.9	44	4.8	39	4.3	61	6.7
Caring for ill people	719	79.0	83	9.1	30	3.3	36	4.0	42	4.6

Regarding changes in students’ well-being, over 75% of the participants reported some worsening level of stress, workload, general well-being, emotional stability, sleep quality, and studies-life balance compared to a face-to-face semester. In the case of eating patterns, this had worsened in some way in 68.5% of the cases. The worst rate was sleep quality (36.4% reported it radically worsened).

However, support levels had only worsened by 14.9% in the case of family support and 25.6% in social support; nearly half of the participants reported that these factors had remained unchanged, Table 5.

**Table 5:** Well-being changes in students during the first academic term of 2020

	Radically worsened		Much worsened		Somewhat worsened		Maintained unchanged		Somewhat improved		Much improved		Radically improved	
	n	%	n	%	n	%	n	%	n	%	n	%	n	%
Stress	252	27.7	334	36.7	228	25.1	50	5.5	28	3.1	14	1.5	4	0.4
Workload	218	24.0	251	27.6	239	26.3	135	14.8	33	3.6	24	2.6	10	1.1
General well-being	280	30.8	284	31.2	222	24.4	68	7.4	29	3.2	16	1.8	11	1.2
Emotional stability	288	31.6	252	27.7	227	24.9	100	11.0	20	2.2	12	1.3	11	1.2
Sleep quality	329	36.2	243	26.7	185	20.3	90	9.9	33	3.6	16	1.8	14	1.5
Eating patterns quality	221	24.3	175	19.2	203	22.3	194	21.3	50	5.5	43	4.7	24	2.6
Family support	16	1.8	41	4.5	78	8.6	443	48.7	97	10.7	129	14.2	106	11.6
Social support	32	3.5	74	8.1	127	14.0	462	50.8	96	10.5	72	7.9	47	5.2
Studies-life balance	221	24.3	242	26.6	239	26.3	131	14.4	42	4.6	21	2.3	14	1.5

### Students’ well-being status related to learning opportunities, resources, social support, and daily activities

**Fig. 2:**
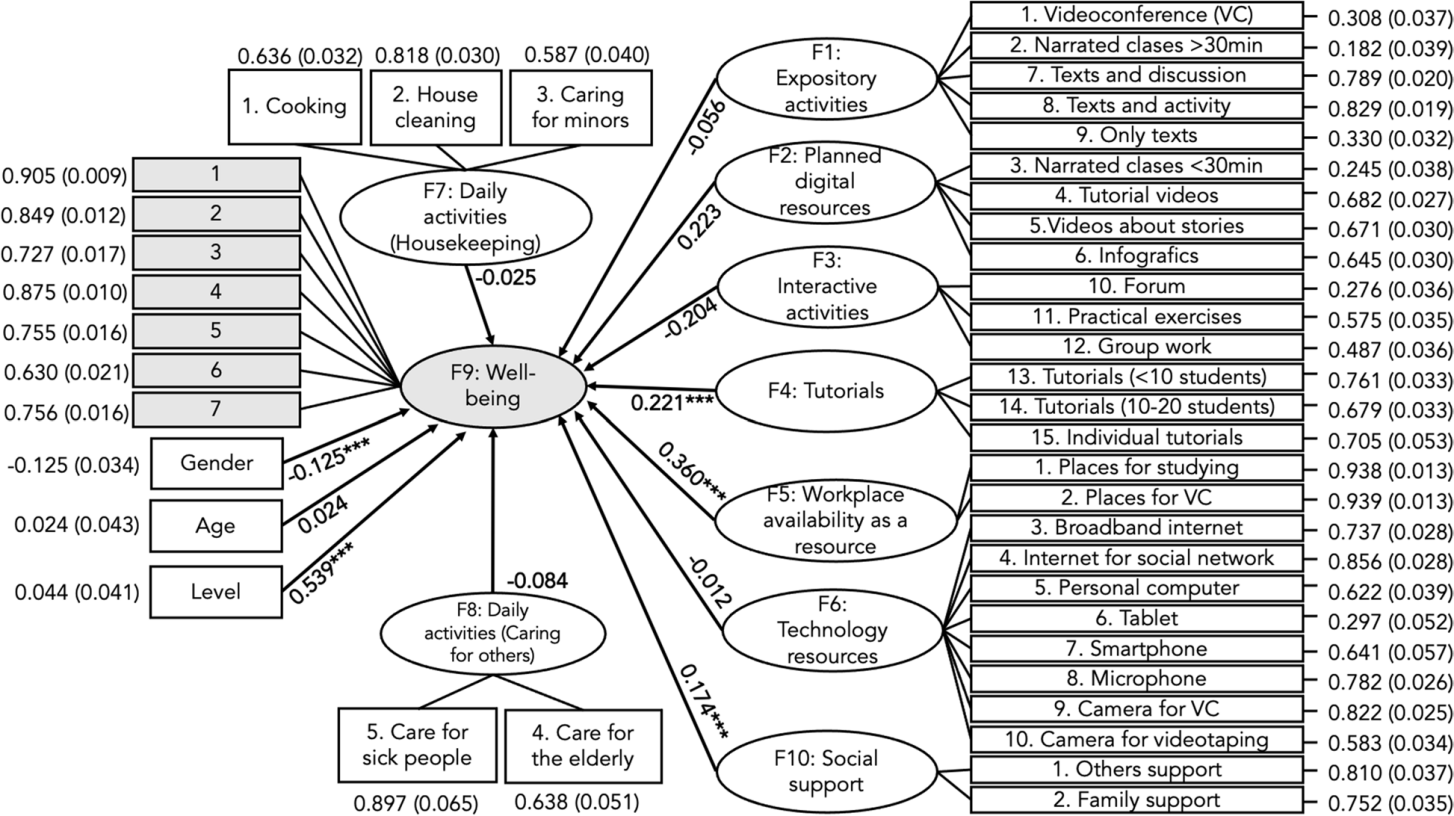
Structural equation modeling with standardized coefficient of well-being in health students

We performed a Structural Equation Modeling method in order to test the relationship that student’s well-being had with learning opportunities (expository activities, planned digital resources, interactive activities, and tutorials), resources (workplace availability and technology resources), social support, and daily activities (housekeeping and caring of others), Fig. 2.

The model showed a χ^2^-test = 2097.282 (df = 771) that was statistically significant (*p* < 0.001) which led to rejecting the model. But due to its power, this test tends to be affected for large samples as analyzed in this study, so we complemented its results with other fit indexes: CFI = 0.945 and TLI = 0.939 showed an adequate fit, and χ2/df = 2.72, RMSEA = 0.043 (90% CI = 0.041–0.046), and SRMR = 0.072 revealed good indexes of fitness, Fig. 2. This means that the proposed model, where well-being is related to learning opportunities, resource availability, daily activities, gender, age, and level, is reasonably consistent with collected data.

Considering learning opportunities, the results expose a direct effect of the students’ participation in tutorial activities in groups with less than 20 students on their well-being (standardized coefficient = 0.221; *p* < 0.001). Also, well-being had a direct effect from workplace availability (standardized coefficient = 0.360; *p* < 0.001).

Social support had a direct effect on positive well-being changes too (standardized coefficient = 0.360; *p* < 0.001), as being women (standardized coefficient = − 0.125; *p* < 0.001) and studying in the last years of the degree (standardized coefficient = 0.539; *p* < 0.001).

## Discussion

The present study provides a detailed view of the reality of undergraduate Health sciences students in Chile, including topics such as resources and living conditions that have received less attention from previous research. The results are based on a heterogeneous sample in terms of geographical area, university, and degree of study. First, in terms of remote teaching activities, the most employed turned out to be the live video conferencing classes, evidencing the overuse of the most employed strategy of traditional face-to-face teaching, the expository course [30–33]. This highly expositive format directly translated to remote teaching, which has also occurred in other countries [6]. It is also evidenced in asynchronous strategies, as another frequently used methodology was the video of more than 30 minutes, a packaged version of the expository class. Moreover, this can be attributed to the emergency nature of the teaching activities employed, where the possibilities of strategically planning the adaptation to online teaching were limited by the lack of time [2].

Nevertheless, it would also be evidence of the system’s attachment to traditional teaching [33], and its insistence on expository strategies that educational experts harshly criticized before the pandemic. Some of the more robust critiques are that expository teaching limits the role of the student to a passive listener and because the retention of learning that it allows is usually poor and makes it difficult for students to transfer them to other contexts [34–36].

Another concern is how frequently students reported teachers providing texts without subsequent activities, as it occurs in more than 85% of the cases, being frequent according to one-third of the students. It reveals that the translation made to emergency remote teaching was, in some cases, using repositories with restricted interaction with the student. Even though teachers participated in training activities to help their adaptation to online education, they seem to neglect the interaction, which can significantly impact learning [37]. However, the lack of time for strategic planning and teacher fatigue amid the pandemic [38] could lead to the temptation to deliver the content as soon as possible to stick to the original volume of content in the learning programs [5].

The most used interaction mechanisms were forums and group work, with significantly lower use of tutorials, even though these personalized meetings could be helpful in detecting technological, educational, or life needs of students in crisis contexts.

Another critical point is how the pandemic has exposed inequity in access to virtual educational resources. Many students did not have the conditions to engage in online teaching actively [28] - causing emergency remote teaching to undermine the principles of quality medical education [37].

Concerning the availability of these resources, we must differentiate technologies used for learning [39] from resources like physical space. It is evident that the smartphone is the most widely available gadget, even more than personal computers. This pattern was detected in the previous studies [8, 40]. and has highlighted the need to design applications to take advantage of this resource [8].

On the other hand, although more than 95% of students reported having access to computers at least once a week, it is questionable whether this availability was sufficient for an on-crisis formative process that continuously depended on access to online resources. It is especially critical when the most vulnerable students lost access to university computer labs due to confinement during the pandemic [16].

Chile, like other countries, invested in providing technologies to the most vulnerable students [16]. But this does not solve the lack of quiet spaces to study and connect at home when required, that a third of the students mentioned. In this sense, the pandemic aggravated inequalities beyond digital inequality.

This inequity also translates to everyday activities. The results show more than half of the students reported that activities such as cooking and cleaning up the house had a high frequency, which is to be expectable given that participants had to stay at home and could not access university food services. Another interesting fact is that more than half of the respondents had to care for a minor, an activity that was daily for almost one-fifth of the participants. Only three-quarters of the sample was free from caring for the elderly and the sick, possibly due to the pandemic confined many students with their families. But it raises the question of whether participants also experienced this reality before the pandemic. How many times did we, as educators, worry about the daily workload experienced by students? How many times did we receive them in the classroom as unburdened subjects?

Moreover, how often were we aware that students were not and adapted our demands to more complex realities than we assumed? In this regard, a survey, an interview, or a short conversation testing these aspects should be a requirement for teaching [16]. It was relevant during the pandemic and will also be necessary for regular education and future crises, because understanding the students’ context is an essential facet of the teaching context, which is key to the didactic knowledge of the content [23].

In terms of levels of well-being, a subset of mental health aspects reports deterioration in most participants: perceived well-being, stress, workload, emotional stability, sleep quality, quality of eating patterns, and study-life balance. These findings are consistent with studies such as that of Czeisler et al. [20], which in June 2020 identified that over 40% of Americans reported some adverse mental health condition, rising to 74.9% in people aged 18–24 years. It is also congruent with studies that demonstrated that the pandemic had caused more significant stress in Health sciences students [9] and university students in general [41–43]. Furthermore, this may be tightly linked to health, economic, and socio-cultural critical conditions of the pandemic [3, 44], the burden of the transition to remote teaching [9], and even the anxiety generated mainly at the beginning of the time of this study because of the lack of understanding the risk of exposure to the virus [45] and the continuous exposure of COVID-19 news in social media [46].

The family and social support levels maintained the same in about half of the participants and improved in 35.6 and 22.9%. These outcomes coincide with the experience reported in other countries [41, 42].

Regarding the relationship of well-being with other variables, it first highlights that those strategies that facilitate direct relationships, such as tutoring, were associated with improved mental health on many factors, primarily perceived well-being and workload. One-on-one, small groups, or medium-sized group tutoring provide the protective effect of human relationships in learning processes but also with the fact that in these interactions, teachers have more opportunities to know the context of students and regulate their academic demands [16]. Furthermore, it could help students meet closer to their classmates, which can help create trusting spaces and promote social bonding. This interaction would be even more convenient for the 2020 and 2021 students who only know university life through screens [14].

Meanwhile, the available resources showed a direct relationship with favorable variations in well-being. The more frequent availability of spaces to study and attend videoconferences was associated with higher well-being. Available spaces are necessary to give the students opportunities to concentrate and pay attention in classes and to help them keep their private life separated from academic life. It coincides with previous research that found that a lack of spaces and resources to study decreases students’ well-being [42].

It also relates to their privacy. The students that put their cams on open the intimacy of their homes to the world, and for many of them, it implies exposing precarious living conditions [47]. But, beyond that, many of them could live in noisy, busy, or chaotic environments, and keeping the cams off could be not only a claim for privacy but the only solution they must stay in class without disturbing it.

Another variable that was significantly related to well-being was gender. Specifically, women showed higher well-being impairment than men. Even in some previous studies in COVID-19 times, gender has not correlated to some forms of well-being such as burnout [48]; in other research, women have shown a higher risk of a general anxiety disorder [49]. It could be associated with gender-biased expectations about women’s performance in Health sciences and degrading treatment that comes with what is not new but remained during the pandemic crisis [50].

Also, in this research, the level taken by students was related to their well-being. First-year students showed higher well-being deterioration than older ones, coinciding with other studies where younger students showed higher emotional exhaustion [48] or anxiety [49] than senior students.

## Conclusions

More than two third of the students reported some kind of well-being deterioration during the first semester of 2020, at the beginning of the COVID-19 pandemic confinement. This deterioration was lower for students who experienced more tutorial activities as teaching strategies from Health schools, those who had a workplace to study more frequently available, and those who reported higher social support. Also, early years students and men experienced more profound deterioration.

## Limitations

Although the present study incorporated six Chilean universities in diverse geographic and social contexts, it is necessary to consider the limitations of the volunteer sampling and the online application employed. Volunteer sampling could generate a more biased sample where personal traits that lead participants to fulfill the survey could generate a more homogeneous survey. In order to diminish its effect, we send the survey twice. So we gave more opportunities to volunteer involved in the study.

On the other hand, online survey could lead to those with more precarious access to the Internet or equipment, especially in the first semester, not having access to the survey, so the difficulties of this group may be underrepresented. However, as confinement forced all students to connect to online teaching, the proportion of offline students is expected to be lower than in previous face-to-face academic years.

Also, our sample had 78.7% women, which could generate a respondent bias, but this is in line with the proportion of women among all the students in Health sciences in Chile. Health sciences have a higher proportion of female students, showing the highest gender gap between all the knowledge areas in enrolled students and the second in graduate students, after Education sciences [51].

### Suggestions for future teaching

This study highlights that medical schools need to promote a combination of asynchronous and synchronous activities for a subject in crisis as well as in regular times. Teachers can promote varied strategies and resources to replace the monotonous, demotivating, and exhausting training routines for students that feel more stressed in a virtual classroom. Moreover, online teaching should also consider the learning context in that each student lives daily in the remote classroom they set up in their home [16, 52]. By doing so, teachers would be more sensible to quickly adapt training activities to those with access difficulties or under multiple responsibilities, either because of the pandemic or their usual living conditions. These are essential to bridge the gap in inequity that a crisis such as the pandemic can pose [53].

It is necessary to remember that the educational crisis and emergency online teaching are not only a technological problem. Above all, it is a pedagogical and psychosocial challenge. During the COVID-19 pandemic, many universities invested in ensuring students’ connectivity [16]. However, it is also necessary to implement preventive strategies to promote students’ and teachers’ well-being and mental health [38]. It includes the need for a sufficient guide to the transition to online learning and the difficulties that students face in remote education, such as difficulties in paying attention, lousy study strategies, or lack of study resources.

Teaching at universities will probably never be the same as before, and shortly online or hybrid teaching will likely be a standard complement to regular training. Even these strategies are likely to become the typical response to future health, climate, and social crisis [2]. Nevertheless, we should not fall back into emergency remote teaching, where the crisis comes as a surprise. We must prepare for the crisis. Health schools must prepare pedagogically and technologically to implement flexible educational innovations that ensure engaged, high-quality, and socially impactful education in the post-Covid era [54].

### Suggestions for future research

We propose future research to continue addressing Health sciences students’ well-being and how it relates to learning opportunities. This research will have the opportunity to compare expository and social involving activities in a face-to-face scenario when pandemic restrictions ended.

Another future line of research is how available quiet places to study are and how they affect the learning process and students’ well-being, mainly in developing countries such as Latin American ones. This variable opens the door to students’ daily living conditions [16] as a key part of the teacher’s didactic knowledge [23].

Additionally, we need to continue researching the psychometric properties of the Well-being Changes Questionnaire. Even if we already evaluated its content validity and internal consistency for this study, we need to add more evidence of its validity related to its response process or its relation to other variables [55], such as current well-being. In this case, tools such as the 5-item World Health Organization Well-Being Index could be an appropriate criterion measure [56].

## Supplementary Information

**Additional file 1.** Remote Teaching Activities Questionnaire

**Additional file 2.** Remote Teaching Activities Questionnaire

## Data Availability

The datasets used and analyzed during the current study are available from the corresponding author on reasonable request.

## Abbreviations

SD: Standard deviation
WLSMV: Weighted Least Square Mean and Variance adjusted
CFI: Comparative Fit Index
TLI: Tucker-Lewis Index
RMSEA: Root Mean Square Error of Approximation
CI: Confidence Interval
SRMR: Standardized Root Mean-Square Residual
df: `Degrees of freedom
χ2/df: Ratio of χ^2^-test to its degrees of freedom.
