# Supplementary material for: Well-being variations on students of health sciences related to their learning opportunities, resources, and daily activities in an online and on-crisis context: a survey-based study
Source: BMC Med Educ. 2023 Jan 18;23:37. doi: 10.1186/s12909-023-04011-y (PMC9848033; doi:10.1186/s12909-023-04011-y)
Supplement: Supplementary file 1 — Additional file 1. Remote Teaching Activities Questionnaire [file 12909_2023_4011_MOESM1_ESM.docx]

SUPPLEMENT 1 - QUESTIONNAIRES IN ENGLISH PROPOSED TRANSLATION

**Remote Teaching Activities Questionnaire**

How often have the subjects you have taken performed the following activities?

|  | Never | Almost never | Sometimes | Almost always | Always |
| --- | --- | --- | --- | --- | --- |
| Videoconference classes | **0** | **1** | **2** | **3** | **4** |
| Long (more than 30 minutes) narrated class videos (with audio) | **0** | **1** | **2** | **3** | **4** |
| Short (less than 30 minutes) narrated lecture videos (with audio) | **0** | **1** | **2** | **3** | **4** |
| Tutorial videos | **0** | **1** | **2** | **3** | **4** |
| Videos about experiences or stories related to the topic | **0** | **1** | **2** | **3** | **4** |
| Infographics or diagrams | **0** | **1** | **2** | **3** | **4** |
| Submission of texts (article, book chapter) with subsequent discussion with the teacher | **0** | **1** | **2** | **3** | **4** |
| Submission of texts (article, book chapter) with subsequent exercise | **0** | **1** | **2** | **3** | **4** |
| Submission of texts (article, book chapter) without subsequent activity | **0** | **1** | **2** | **3** | **4** |
| Forums | **0** | **1** | **2** | **3** | **4** |
| Practical exercises | **0** | **1** | **2** | **3** | **4** |
| Group work | **0** | **1** | **2** | **3** | **4** |
| Tutorial meetings with the teacher in small groups (less than 10) | **0** | **1** | **2** | **3** | **4** |
| Tutorial meetings with the teacher in medium-sized groups (between 10 and 20 students) | **0** | **1** | **2** | **3** | **4** |
| Tutorial meetings with the teacher in individual groups | **0** | **1** | **2** | **3** | **4** |

**Learning Resources Availability Questionnaire**

How often do you count on the following resources during this semester? Consider also the resources provided by cell phones.

|  | Never | At least once a month | At least once a week | A few days each week | At least once a day | Whenever required |
| --- | --- | --- | --- | --- | --- | --- |
| Quiet spaces for studying | **0** | **1** | **2** | **3** | **4** | **5** |
| Quiet spaces to participate in videoconferences | **0** | **1** | **2** | **3** | **4** | **5** |
| Broadband Internet access | **0** | **1** | **2** | **3** | **4** | **5** |
| Internet access for social networking only | **0** | **1** | **2** | **3** | **4** | **5** |
| Computer | **0** | **1** | **2** | **3** | **4** | **5** |
| Tablet | **0** | **1** | **2** | **3** | **4** | **5** |
| Smartphone | **0** | **1** | **2** | **3** | **4** | **5** |
| Microphone for videoconferencing | **0** | **1** | **2** | **3** | **4** | **5** |
| Camara for videoconferencing | **0** | **1** | **2** | **3** | **4** | **5** |
| Camara for videorecording | **0** | **1** | **2** | **3** | **4** | **5** |

**Daily Life Activities Questionnaire**

What type of work has this semester performed daily?

|  | Never | Few times  by month | At least  once a week | Some days  each week | Every day |
| --- | --- | --- | --- | --- | --- |
| Cooking | **0** | **1** | **2** | **3** | **4** |
| House cleaning | **0** | **1** | **2** | **3** | **4** |
| Caring for minors | **0** | **1** | **2** | **3** | **4** |
| Caring for the older adults | **0** | **1** | **2** | **3** | **4** |
| Caring for ill people | **0** | **1** | **2** | **3** | **4** |

**Well-being Changes Questionnaire**

How much have the following factors varied compared to an average academic year?

|  | Radically worsened | Much worsened | Somewhat worsened | Maintained unchanged | Somewhat improved | Much improved | Radically improved |
| --- | --- | --- | --- | --- | --- | --- | --- |
| My stress levels | **-3** | **-2** | **-1** | **0** | **1** | **2** | **3** |
| My workload | **-3** | **-2** | **-1** | **0** | **1** | **2** | **3** |
| My sense of well-being (e.g., sense of tranquility, fullness) | **-3** | **-2** | **-1** | **0** | **1** | **2** | **3** |
| My emotional stability (e.g. absence of mood swings, the predominance of positive emotional states) | **-3** | **-2** | **-1** | **0** | **1** | **2** | **3** |
| The quality of my sleep (e.g. feeling rested after sleep, feeling that I slept well) | **-3** | **-2** | **-1** | **0** | **1** | **2** | **3** |
| The quality of my eating patterns (e.g., eating healthy foods, following established schedules, etc.). | **-3** | **-2** | **-1** | **0** | **1** | **2** | **3** |
| The family support I have | **-3** | **-2** | **-1** | **0** | **1** | **2** | **3** |
| The social support I have | **-3** | **-2** | **-1** | **0** | **1** | **2** | **3** |
| The compatibility between my academic activities and activities in other areas of my personal life (e.g. self-care, rest, leisure, housework). | **-3** | **-2** | **-1** | **0** | **1** | **2** | **3** |
