# Supplementary material for: Well-being variations on students of health sciences related to their learning opportunities, resources, and daily activities in an online and on-crisis context: a survey-based study
Source: BMC Med Educ. 2023 Jan 18;23:37. doi: 10.1186/s12909-023-04011-y (PMC9848033; doi:10.1186/s12909-023-04011-y)
Supplement: Supplementary file 2 — Additional file 2. Remote Teaching Activities Questionnaire [file 12909_2023_4011_MOESM2_ESM.docx]

SUPPLEMENT 2 - QUESTIONNAIRES IN SPANISH ORIGINAL VERSION

**Cuestionario de Actividades en Docencia Remota**

¿Con qué frecuencia las asignaturas que ha cursado en este semestre han realizado las siguientes actividades?

|  | Never | Almost never | Sometimes | Almost always | Always |
| --- | --- | --- | --- | --- | --- |
| Clases por videoconferencia | **0** | **1** | **2** | **3** | **4** |
| Videos de clases narradas (con audio) largos (más de 30 minutos) | **0** | **1** | **2** | **3** | **4** |
| Videos de clases narradas (con audio) cortos (menos de 30 minutos) | **0** | **1** | **2** | **3** | **4** |
| Videos tutoriales | **0** | **1** | **2** | **3** | **4** |
| Videos sobre experiencias o historias relacionadas con el tema | **0** | **1** | **2** | **3** | **4** |
| Infografias o esquemas | **0** | **1** | **2** | **3** | **4** |
| Envío de textos (artículo, capítulo de libro) con discusión posterior con el docente | **0** | **1** | **2** | **3** | **4** |
| Envío de textos (artículo, capítulo de libro) con ejercicio posterior | **0** | **1** | **2** | **3** | **4** |
| Envío de textos (artículo, capítulo de libro) sin actividad posterior | **0** | **1** | **2** | **3** | **4** |
| Foros | **0** | **1** | **2** | **3** | **4** |
| Ejercicios prácticos | **0** | **1** | **2** | **3** | **4** |
| Trabajos grupales | **0** | **1** | **2** | **3** | **4** |
| Reuniones de tutorías con el docente en pequeños grupos (menos de 10) | **0** | **1** | **2** | **3** | **4** |
| Reuniones de tutorías con el docente en grupos medianos (entre 10 y 20 estudiantes) | **0** | **1** | **2** | **3** | **4** |
| Reuniones de tutorías con el docente individuales | **0** | **1** | **2** | **3** | **4** |

**Cuestionario de Disponibilidad de Recursos para el Aprendizaje**

¿Con qué frecuencia cuenta con los siguientes recursos durante este semestre? Considere también los recursos que aportan los teléfonos móviles.

|  | Nunca | Al menos una vez al mes | Al menos una vez por semana | Algunos días a la semana | Al menos una vez al día | Cada vez que lo requiera |
| --- | --- | --- | --- | --- | --- | --- |
| Espacios tranquilos para estudiar | **0** | **1** | **2** | **3** | **4** | **5** |
| Espacios tranquilos para participar en videoconferencias | **0** | **1** | **2** | **3** | **4** | **5** |
| Acceso a internet banda ancha | **0** | **1** | **2** | **3** | **4** | **5** |
| Acceso a internet sólo para redes sociales | **0** | **1** | **2** | **3** | **4** | **5** |
| Computador | **0** | **1** | **2** | **3** | **4** | **5** |
| Tablet | **0** | **1** | **2** | **3** | **4** | **5** |
| Teléfono inteligente | **0** | **1** | **2** | **3** | **4** | **5** |
| Micrófono para videoconferencias | **0** | **1** | **2** | **3** | **4** | **5** |
| Cámara para videoconferencias | **0** | **1** | **2** | **3** | **4** | **5** |
| Cámara para grabar videos | **0** | **1** | **2** | **3** | **4** | **5** |

**Cuestionarios de Actividades de la Vida Diaria**

¿Qué tipo de labores ha realizado cotidianamente este semestre?

|  | Nunca | Pocas veces al mes | Al menos una vez por semana | Varias veces por semana | Todos los días |
| --- | --- | --- | --- | --- | --- |
| Cocinar | **0** | **1** | **2** | **3** | **4** |
| Realizar el aseo del hogar | **0** | **1** | **2** | **3** | **4** |
| Cuidar a menores de edad | **0** | **1** | **2** | **3** | **4** |
| Cuidar adultos mayores | **0** | **1** | **2** | **3** | **4** |
| Cuidar personas enfermas | **0** | **1** | **2** | **3** | **4** |

**Cuestionario de Cambios en el Bienestar**

¿Cuánto han variado los siguientes factores en comparación con un año académico normal?

|  | Radically worsened | Much worsened | Somewhat worsened | Maintained unchanged | Somewhat improved | Much improved | Radically improved |
| --- | --- | --- | --- | --- | --- | --- | --- |
| Mis niveles de estrés | **-3** | **-2** | **-1** | **0** | **1** | **2** | **3** |
| Mi carga de trabajo en comparación a un semestre presencial | **-3** | **-2** | **-1** | **0** | **1** | **2** | **3** |
| Mi sensación de bienestar (p.e. sensación de tranquilidad, plenitud) | **-3** | **-2** | **-1** | **0** | **1** | **2** | **3** |
| Mi estabilidad emocional (p.e. ausencia de cambios bruscos de humor, predominancia de estados emocionales positivos) | **-3** | **-2** | **-1** | **0** | **1** | **2** | **3** |
| La calidad del sueño (p.e. sentirse descansado después de dormir, sentir que se durmió bien) | **-3** | **-2** | **-1** | **0** | **1** | **2** | **3** |
| La calidad de mis patrones de alimentación (p.e. consumo de alimentos saludables, seguir horarios establecidos, etc.) | **-3** | **-2** | **-1** | **0** | **1** | **2** | **3** |
| El apoyo familiar con el que cuento | **-3** | **-2** | **-1** | **0** | **1** | **2** | **3** |
| El apoyo social con el que cuento | **-3** | **-2** | **-1** | **0** | **1** | **2** | **3** |
| La compatibilización entre mis actividades académicas con actividades de otras áreas de mi vida personal (p.e. autocuidado, descanso, ocio, labores domésticas) | **-3** | **-2** | **-1** | **0** | **1** | **2** | **3** |
